# Review of the Canadian Nontuberculous Mycobacterial Disease Landscape—Challenges and Opportunities

**DOI:** 10.3390/tropicalmed10120328

**Published:** 2025-11-24

**Authors:** Sepideh Vahid, Marie Yan, Shannon Lee Turvey

**Affiliations:** 1 Department of Pharmaceutical Sciences, Vancouver General Hospital, Vancouver, BC V5Z 1M9, Canada; 2 Division of Respiratory Medicine, Department of Medicine, Faculty of Medicine and Dentistry, University of British Columbia, Vancouver, BC V5Z 1M9, Canada; 3 Division of Infectious Diseases, Department of Medicine, Faculty of Medicine and Dentistry, University of British Columbia, Vancouver, BC V5Z 3J5, Canada

**Keywords:** nontuberculous mycobacteria, *Mycobacterium avium* complex, *Mycobacterium abscessus*, bronchiectasis, antimicrobial resistance, Canada

## Abstract

The incidence and prevalence of nontuberculous mycobacterial (NTM) disease are rising. This narrative review examines the evolution of NTM disease trends over the past four decades, in Canada and globally, encompassing changing epidemiology, shifting treatment paradigms, and emerging antimicrobial resistance patterns. Challenges to NTM treatment are explored, and novel and investigational therapies are summarized. Key themes include a significant increase in NTM disease incidence, temporal shifts in the dominant species causing human infections, evolution from single-drug to multi-drug treatment approaches, and growing concerns regarding macrolide resistance. The substantial challenges with treatment tolerability, effectiveness, and access are outlined. This review synthesizes data from multiple sources, including peer-reviewed literature, clinical trials, and public health databases, to provide a comprehensive understanding of the changing NTM disease landscape in Canada and more broadly. There is a need for expanded surveillance, continued innovation, and a multidisciplinary approach to NTM management.

## 1. Introduction

Nontuberculous mycobacteria (NTM) is an umbrella term for all mycobacterial species other than *Mycobacterium tuberculosis* complex (MTBC) and *M. leprae*. NTM are a diverse and ubiquitous group of environmental organisms that can cause pulmonary and extrapulmonary disease in susceptible hosts. In this narrative review, we outline trends in NTM disease epidemiology with a Canadian lens. We explore the evolution of NTM treatment, patterns of in vitro resistance, challenges facing NTM treaters and patients living with NTM disease, and opportunities and future directions in the NTM landscape.

## 2. Historical Context and Epidemiology of NTM Disease in Canada

### 2.1. NTM Pulmonary Disease

In parallel with global trends, the prevalence of NTM pulmonary disease (NTM-PD) in Canada has risen over the past four decades [1,2,3,4]. Unlike MTBC, NTM species generally do not exhibit person-to-person transmission (although some cases of person-to-person transmission of *M. abscessus* in the context of cystic fibrosis have been documented) [5]. Since NTM species are commonly found in the environment (e.g., water supply and soil), isolation of NTM from non-sterile sites could represent colonization or contamination and is not always indicative of disease [5,6,7,8,9]. Accordingly, true disease incidence is difficult to estimate, given that NTM-PD is not a reportable condition in most regions and not all pulmonary isolates necessarily represent disease [7,10]. Therefore, epidemiologic data are patchy both globally and regionally. However, an overwhelming majority of published studies have reported an increase in NTM infection and NTM-PD over time [4,11,12,13,14,15,16,17]. In Queensland, Australia, where NTM disease is notifiable, a substantial increase was observed over 10 years, from 672 cases in 2012 [4] to 1490 cases in 2022 [18]. The reasons for this increase are uncertain and likely multifactorial. In part, the rising global importance of NTM-PD can plausibly be attributed to increased case-finding due to increased use of high-resolution chest imaging and improved sensitivity of in vitro mycobacterial culture techniques [19,20,21]. Increased awareness of NTM-PD among providers could also be a contributing factor, as microbiologic diagnosis requires a high level of diagnostic suspicion and appropriate testing via sputum sampling or bronchoscopy [5]. Shifting demographics have led to rising numbers of susceptible hosts—including advanced age, immunocompromised individuals, and structural lung disease such as chronic obstructive pulmonary disease and bronchiectasis [22,23]. Environmental factors may also play an important role in the rising prevalence of NTM disease [5,24]. Epidemiological studies have found a correlation between NTM infection risk and average temperature, as well as levels of precipitation and rates of water cycling [25]. Urbanization and global climate trends may lead to increased environmental NTM exposure through changing temperature, humidity, and extreme weather events that put people at risk of soil and water exposure [6,10,26,27]. Other postulated contributors are increasing antibiotic use, driving a shift in lung microbiome, and potentially falling rates of disease due to MTBC in some populations [28,29].

**Figure 1:**
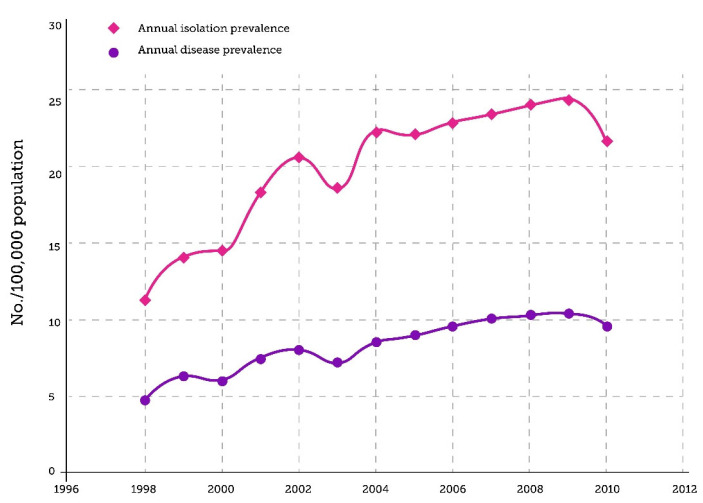
Annual Isolation Prevalence and Disease Prevalence per 100,000 Persons of Pulmonary Nontuberculous Mycobacteria, Ontario, Canada, 1998–2010. (Adapted from Marras et al., 2013 [1]).

Epidemiologic data specific to the Canadian population mostly come from Ontario, with sparse data from other provinces/territories, In the early 1980s, NTM disease prevalence in North America appeared to be relatively low [30]. During the 1980s and 1990s, NTM disease was primarily recognized in immunocompromised patients, particularly people living with human immunodeficiency virus (HIV) infection. Disease recognition outside this population was limited by laboratory diagnostic capabilities, lack of standardized identification methods, low clinical awareness, and a focus on MTBC as the primary mycobacteriosis of concern. A retrospective review of NTM isolates from 1991 to 1995 at the British Columbia Centre for Disease Control found an overall incidence rate of pulmonary NTM disease in non-immunosuppressed hosts of 0.000063 per year, with no statistically significant trend observed over the 4-year study period [31]. However, retrospective data from Ontario indicate a substantial rise in NTM disease prevalence in recent decades [1,29,32]. Between 1998 and 2010, five-year prevalence increased from 29.3 cases/100,000 persons to 41.3/100,000 (Figure 1). A further 2.5-fold increase in the prevalence of *M. avium* was observed in Ontario between 2010 and 2020 [29]. This mirrors global trends, which suggest a genuine surge in disease occurrence rather than merely detection bias [33,34].

While there are over 190 distinct NTM species, not all are significant causes of human disease [10,35]. The most common NTM species affecting Canadians are *M. avium* and *M. intracellulare*, which are members of the *M. avium* complex (MAC), as well as *M. abscessus* and *M. xenopi*. According to available data, *M. avium* is the most prevalent species isolated in Ontario, accounting for between 69.2% to 73.1% of NTM cases in 2020 [29,36]. *M. intracellulare* (a member of the MAC) and *M. abscessus* (MAB) follow as the next most common species. In British Columbia, MAC accounted for 77% of NTM isolates from pulmonary specimens between 1990 and 2006 [37,38]. There is often geographic variation in the prevalence of these species [39]. For example, *M. xenopi* is relatively common in Ontario, but infrequently encountered in other regions of Canada [29,40]. Certain species, such as *M. malmoense*, which is common in Northern Europe, seem to be confined to specific regions in the world and are rarely seen in Canada.

Age is a significant risk factor for NTM disease [41]. In Ontario, the age-standardized prevalence ratio for all NTM species combined increased from 0.05 in the youngest age group to 4.46 in the oldest age group [29]. The prevalence of MAC pulmonary disease (MAC-PD) in Ontario showed a stark contrast between age groups: 1 per 100,000 people for those younger than 50, versus 48 per 100,000 people for those 80 and older [42]. Additionally, older Canadians have been disproportionately impacted by shifts in disease prevalence over time. The prevalence of MAC-PD in Ontario in people over 50 increased from 11.9 to 18.6 per 100,000 in people over 50 years old between 2003 and 2008 [42]. Furthermore, a shift in gender distribution has been observed from male predominance to female predominance of NTM-PD, particularly in older individuals [43]. This has resulted in the largest disease burden of NTM-PD now observed in the older female demographic [6,32]_._ The variation in prevalence across age groups, as well as differential susceptibility to adverse effects of medications, highlights the importance of age-specific considerations in diagnosing and managing NTM infections.

### 2.2. Extrapulmonary NTM Disease

While pulmonary disease is the most common form of NTM disease worldwide, NTM can also involve extrapulmonary sites or develop into disseminated disease. Commonly affected extrapulmonary sites include skin and soft tissue, bone and joint, and lymph nodes [44]. Like lung disease, the prevalence of disseminated and extrapulmonary NTM disease have also been increasing over time [45,46]. Two retrospective studies found a rise in cutaneous NTM disease in Alberta, Canada, as well as a rise in cervicofacial NTM disease in children in Montreal, Quebec [47,48]. The increase in popularity of cosmetic surgical procedures has also driven an increase in skin and soft tissue NTM disease [49].

Disseminated NTM infections can have a long incubation period and generally present with nonspecific symptoms, making diagnosis challenging [50]. This can lead to underreporting and delayed treatment initiation [50]. Acquired immune suppression, such as biologic immune modulators and immune suppression in organ transplantation, is an important driver of propensity to mycobacterial and other forms of infection, and disseminated NTM disease is rarely seen in non-immunosuppressed individuals [5]. Recently, autoimmune-mediated susceptibility via the production of anti-IFN-γ autoantibodies was also identified as an important risk factor in some regions [51].

Healthcare contact has been implicated in cases of disseminated NTM disease [50]. A notable example is the recent global outbreak of disseminated *M. chimaera* linked to contaminated Sorin (Stöckert^®^) 3T heater-cooler units utilized in open heart surgery, with whole genome sequencing (WGS) supporting a point source [52,53]. The first cardiac surgeries to be associated with disseminated *M. chimaera* infection were originally performed in 2006 [54]. However, the latency period was prolonged (median time to presentation 16 months, maximum reported incubation time 12 years), the clinical presentation was nonspecific, and initially the syndrome and epidemiologic linkage were unknown [55]. The first cases were not reported until 2013, and cases continued to be identified for some time after the contaminated heater-cooler units were no longer in use [55,56,57]. In Canada, the first 2 cases of disseminated *M. chimaera* post cardiovascular surgery were identified in 2016 in Montreal [58]. Subsequently, cases were identified at multiple Canadian sites. Mortality rates in this outbreak were high, with mortality estimates ranging from 45% to >65% and one systematic review reported median survival of two years after treatment start or diagnosis [55,59]. An important takeaway from this outbreak was that surgical intervention (either removal or exchange of prosthetic material) was associated with statistically better survival [55].

## 3. Evolution of NTM Therapies

NTM disease treatment has evolved significantly over time, shifting from monotherapy to more complex combination antibiotic regimens. This progression reflects growing awareness of the capacity of NTM species to develop resistance to antimicrobials while on therapy and the need for more effective NTM treatment strategies. The first significant advancement came in 1990 when the American Thoracic Society recommended a four-drug regimen for MAC consisting of isoniazid, rifampin, ethambutol, and streptomycin [10,60]. This marked a crucial shift towards combination therapy, recognizing the need for a multi-pronged approach to combat NTM infections effectively. Another breakthrough occurred in 1997 with the incorporation of macrolides (clarithromycin or azithromycin) into the treatment regimen [10,61]. This was informed by a randomized controlled trial which demonstrated superiority of a three-drug regimen consisting of clarithromycin, ethambutol and rifabutin over a four-drug regimen consisting of clofazimine, ethambutol, rifabutin, and ciprofloxacin in patients with advanced HIV infection and disseminated MAC [62]. In this Canadian HIV Trials Network study, the macrolide-based regimen cleared MAC mycobacteremia more rapidly and more often, and survival rates were higher in this group [62]. Routine use of a macrolide in MAC treatment regimens significantly improved treatment outcomes, with studies showing conversion rates between 78% and 92% and lower relapse rates [10]. Macrolides have since become a cornerstone of NTM antimicrobial therapy, particularly for MAC infections. Today, the standard treatment for most NTM infections involves a combination of at least three antibiotics taken over an extended period, typically 18 to 24 months [10,63]. Although rifamycins are often used in MAC treatment, a retrospective Calgary study found that clofazimine use was associated with significantly higher rates of sputum culture conversion than rifampin use (100% vs. 71%; *p* = 0.002) [64].

More recently, there has been interest in whether a two-drug regimen may be as effective as a three-drug regimen for MAC treatment while being better tolerated. However, effective protection against emerging resistance remains a concern. National Institutes of Health guidelines for the treatment of opportunistic infections in people living with HIV suggest a two-drug regimen of azithromycin and ethambutol for some patients, with addition of a third and potentially a fourth drug in those with severe disease or if effective antiretroviral therapy is not being initiated [65]. A recent meta-analysis found no difference in outcome between a two-drug regimen consisting of macrolide and ethambutol and a three-drug regimen for treatment of pulmonary MAC [66]. In this meta-analysis, use of a 2-drug regimen to treat MAC was associated with an increased rate of emergent macrolide resistance [66]. Another single-centre retrospective outcome analysis of 108 patients treated with a two-drug regimen of daily azithromycin and ethambutol for pulmonary MAC, regardless of phenotype (nodular bronchiectatic vs. fibrocavitary), found that 2/18 patients with refractory disease developed new macrolide resistance [67]. A multicentre randomized pragmatic clinical trial (NCT03672630) is currently ongoing to compare the outcomes of 2-drug vs. 3-drug antimicrobial therapy in the treatment of MAC-PD.

Specific treatment recommendations for each NTM species are beyond the scope of this article and can be found elsewhere [6,68]. In general, antimicrobial therapy varies depending on NTM classification (rapidly growing vs. slow growing), specific species, in vitro antimicrobial susceptibility testing for key drugs, and the clinical context [7,10]. Regardless of species, treatment courses are typically prolonged.

## 4. Role of Non-Medication Treatment in the Management of NTM Disease

The management of NTM pulmonary disease requires a multimodal approach, integrating both pharmacological and non-pharmacological strategies to optimize patient care. These strategies include implementing effective airway clearance, optimizing nutrition, minimizing gastroesophageal reflux, and reducing environmental NTM exposure [69]. Airway clearance plays an important role in the management of NTM-PD, particularly due to the association of the disease with bronchiectasis, which is an irreversible condition characterized in part by impaired airway clearance [70]. Effective airway clearance—which can involve various physical techniques, devices, and mucoactive agents tailored to each patient—facilitates the removal of excess inflammatory mucus and bacteria from bronchiectatic airways, to reduce symptoms and prevent disease progression [71,72]. Studies have shown that adherence to airway clearance techniques is associated with improvement in subjective (cough, sputum production) and objective (total lung capacity) markers of disease control [25,70,73]. In a small subset of patients with mild NTM-PD disease, use of airway clearance techniques alone may result in sputum culture conversion from positive to negative [70,74,75].

Surgery is another adjunctive modality that can be utilized in NTM treatment, both for pulmonary and for extra-pulmonary NTM disease [76,77,78,79,80]. Surgical resection for patients with focal pulmonary disease is associated with a high likelihood of sputum culture clearance (81% in one retrospective review of 70 patients), although selection bias may be relevant here [81]. Surgery has a low mortality rate but can be associated with significant morbidity, particularly bronchopleural fistula. It is important to recognize that surgery for NTM-PD does not negate the need for antibiotic treatment, and therefore, surgery is not a panacea for patients with antibiotic intolerance or adverse effects. For NTM-PD, surgery should be considered in the following scenarios for carefully selected patients where surgical expertise exists: 1. Patients with poor response to antibiotic treatment, based on persistent symptoms or persistently positive mycobacterial sputum cultures despite optimized medical management; 2. Patients with focal lung disease or residual structural lesions that can be surgically resected; 3. Refractory hemoptysis [79,82].

For extra-pulmonary NTM disease, surgery is critical in a broader subset of patients [44,83]. For patients with NTM skin and soft tissue infection following cosmetic procedures or for those with osteoarticular NTM disease, debridement and removal of infected prosthetic material is essential for cure [49,84,85,86]. In patients receiving peritoneal dialysis who develop NTM peritonitis, peritoneal dialysis catheter removal is recommended [87]. In central line-associated bloodstream infection secondary to NTM, removal of the central line is a prerequisite for clearance of the disease [88]. NTM cervical lymphadenitis is typically seen in the pediatric population and, with the exception of people living with HIV infection, rarely affects adults [44]. For pediatric patients with NTM lymphadenitis, resection of the involved lymph nodes is associated with a higher likelihood of resolution of infection and is generally curative [44].

## 5. Challenges in NTM Treatment

**Figure 2:**
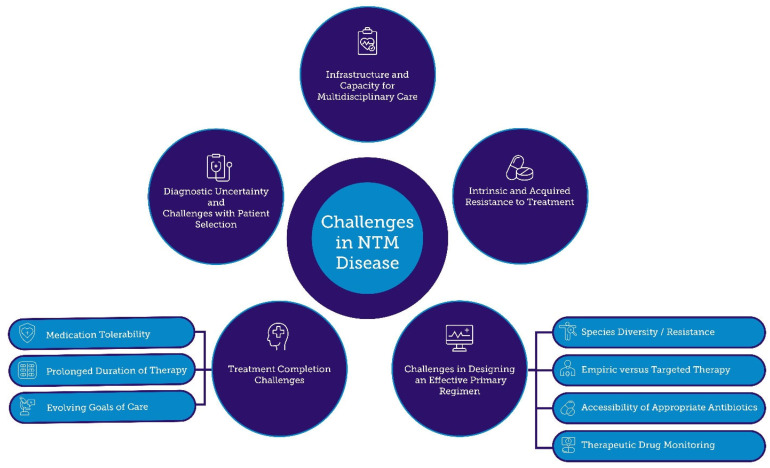
Major Challenges in the Diagnosis and Management of Nontuberculous Mycobacterial (NTM) Disease.

In the following section, we focus on the principal hurdles in NTM treatment—spanning diagnostic and regimen-design challenges, antimicrobial resistance, and issues of tolerability and adherence—that collectively undermine optimal patient outcomes. These complex factors and the interplay between them are visually summarized in Figure 2.

### 5.1. Challenges with Patient Selection

The treatment of NTM-PD is multifaceted and complex. In contrast to extra-pulmonary NTM disease, where treatment is always indicated, not all patients with NTM-PD will benefit from antibiotic therapy. For NTM-PD, guidelines recommend continuing antibiotic treatment for most species (except *M. kansasii*) for at least 12 months following sputum culture conversion. The median duration of treatment is typically around 18 months, although many patients may remain on treatment for years [7,10]. However, the antibiotics are often difficult to tolerate and can be associated with many adverse effects [89,90]. Early interruption or discontinuation of treatment is common, with one retrospective multicentre cohort study reporting therapy discontinuation due to adverse drug events in 35% of patients, with a median time to discontinuation of 32 days [89]. Even with long treatment duration and multidrug regimens, rates of microbiological cure remain suboptimal (~60% and 45% for MAC and *M. abscessus*, respectively), and recurrence is common [91]. With the notable exception of *M. kansasii* (where relapse occurs approximately 6–7% of the time), 10–60% of patients with NTM-PD who clear their sputum cultures will experience relapse or reinfection within 6–12 months of treatment completion [92,93,94,95]. Over time, decreased sputum conversion rates among patients in Ontario, Canada, being treated for *M. avium* have been observed [96].

A recent retrospective single-centre study of 434 patients in Montreal with at least one NTM isolated from a respiratory site found that 38/68 patients who met guideline-based diagnostic criteria for NTM-PD were treated with antibiotics [97]. This study found a high rate of poor outcome regardless of the decision to treat. 21/38 patients who started treatment did not complete treatment and 13/21 patients who did not complete treatment died during treatment or within 12 months of treatment initiation [97]. Rate of adverse effects on therapy leading to regimen change or treatment discontinuation was 45% [97]. In this study, acknowledging the limitations of the retrospective design, treatment did not appear to impact survival or modify disease course [97].

Given the potential risks of antibiotics, the uncertainty of clinical benefit, and low rates of “cure”, antibiotic treatment is not always recommended. In many cases with stable and/or mild pulmonary disease, antibiotics are not necessary, and patients may be supported with nonpharmacological management. However, it can be challenging to determine which patients would benefit from antibiotic treatment, and currently, there are no clinical tools or biomarkers that can reliably predict disease progression or treatment responsiveness. As it stands, the decision to initiate antimicrobial therapy should involve a patient-centred discussion, carefully weighing the potential benefits and risks and considering patient preferences and goals of care.

### 5.2. Resistance to Antibiotic Treatment

Several intrinsic microbial features hinder the efficacy of medications and complicate the discovery of novel NTM agents. As species that evolved to resist environmental insults, NTMs’ slow growth, ability to enter a dormancy phase, their lipid-rich outer membrane that limits drug permeation, biofilm formation on alveolar surfaces, and intracellular survival within phagocytes all protect against antibiotic exposure and host immunity [9,98]. These conserved characteristics render NTM relatively resistant to treatment and drive the need for prolonged courses of multidrug therapy. Additionally, inducible resistance mechanisms—such as upregulated efflux pumps, antibiotic-modifying enzymes, and mutation-driven resistance—further complicate drug efficacy. Overall, antimicrobial resistance represents a significant challenge to treatment and, in some cases, a major determinant of outcomes.

In Canada, in vitro antimicrobial susceptibility testing is not routinely performed on all clinical NTM isolates. This can be requested and, in Canada, is typically performed at the National Microbiology Lab. For some drugs, established clinical breakpoints recommended by the Clinical and Laboratory Standards Institute do not exist, and only the minimum inhibitory concentration (MIC) is reported [99]. With a few exceptions, in vitro antimicrobial susceptibility testing has not been demonstrated to be associated with clinical outcomes in the treatment of NTM pulmonary disease [100]. However, baseline in vitro susceptibility testing is recommended for isolates felt to be clinically important [6], and most NTM disease experts utilize in vitro susceptibility results to inform regimen selection.

There are some cases in which in vitro antimicrobial susceptibility testing has been shown to correlate with clinical outcomes. Macrolide resistance in MAC is strongly associated with worse clinical outcomes, including higher mortality [101,102,103,104]. Macrolide-resistant MAB also have worse clinical outcomes [105,106]. Amikacin susceptibility predicts clinical response of MAC disease to amikacin-based treatment [107,108]. For *M. kansasii*, rifampin susceptibility predicts clinical response to rifampin-based treatment. For some NTM species such as *M. marinum* and *M. ulcerans*, susceptibility is generally predictable and emergent resistance uncommon, and antimicrobial susceptibility testing is challenging and not always clinically useful [44,109]. However, resistance has been observed, and susceptibility testing should be considered where feasible in severe or refractory cases [110,111].

Increasingly, molecular methods of antimicrobial susceptibility testing may have utility in early and accurate detection of resistance among clinical NTM isolates [112,113]. Molecular assays like the GenoType NTM-DR line probe assay can aid in the rapid detection of resistance, allowing for more tailored and effective treatment regimens [112,113]. Despite their relevance to predicting clinical outcome, specific quantitative data on resistance rates for clarithromycin, amikacin, and rifampin among non-tuberculous mycobacteria (NTM) species can vary by region and study and are not collected systematically. However, some retrospective data are available for the most important antibiotics used in NTM treatment.

#### 5.2.1. Macrolide Resistance

Macrolides (clarithromycin and azithromycin) are cornerstone drugs for NTM disease treatment. Macrolide resistance significantly complicates treatment and is associated with a higher risk of treatment failure (approximately 70% in macrolide-susceptible MAC to approximately 20–40% in macrolide-resistant MAC) [103,114]. A primary goal of treatment for macrolide-susceptible MAC is to prevent the emergence of macrolide resistance, and ethambutol plays a pivotal role in protecting the macrolide [6,115,116]_._ Macrolide monotherapy (including for chronic lung conditions such as chronic obstructive pulmonary disease and bronchiectasis) is a risk factor for macrolide-resistant MAC, and screening for NTM prior to initiation of macrolide maintenance therapy is a guideline recommendation [102,104,117,118,119]. Additionally, a prior history of NTM treatment is an important predictor of emergent macrolide resistance [120]. In 2022 in British Columbia, macrolide resistance among MAC isolates which were tested was reported to be 10% [121]. However, as rates of NTM disease rise and NTM-PD is increasingly recognized and treated, it will be interesting to see whether local resistance data will change. Some studies have reported that resistance to macrolides among NTM, particularly MAC, has been increasing. In China, one study reported 21% macrolide resistance among 244 *M. intracellulare* isolates, with MIC50 and MIC90 values of 8 and 64 mg/L, respectively [122].

MAB is much more likely than MAC to be resistant to macrolides in vitro. In Israel, clarithromycin was effective against 97% of MAC isolates, but only 39% of MAB isolates were susceptible [123]. However, resistance rates vary depending on MAB subspecies [124]. Susceptibility to macrolides, particularly inducible resistance, is an important predictor of MAB treatment outcome [124]. *M. abscessus* subspecies *abscessus* evinces constitutive (*rrl* gene) or inducible (*erm41* gene) macrolide resistance over 50% of the time [122]. Inducible resistance (at day 14) is more common and is due to the presence of a functional *erm41* gene encoding a methylase enzyme responsible for macrolide resistance [125]. *M. abscessus* subspecies *massiliense* is more likely to retain macrolide susceptibility than *M. abscessus* subspecies *abscessus* [122,125]. This is because *M. abscessus* subspecies *massiliense* has a truncated non-functional *erm41* gene [125]. *M. abscessus* subspecies *bolletii* exhibits innate macrolide resistance [125].

#### 5.2.2. Aminoglycoside Resistance

Resistance to aminoglycosides (streptomycin, amikacin, tobramycin) is also found among both slow growing and rapidly growing NTM. The clinical breakpoints for amikacin in MAC vary depending on the mode of delivery. For amikacin liposome inhalation suspension (ALIS), the breakpoint for resistance recommended by the Clinical and Laboratory Standards Institute is ≥128 µg/mL [7,126]. The breakpoints for intravenous amikacin for MAC, as currently recommended by the Clinical and Laboratory Standards Institute, are: ≤16 μg/mL for susceptible, 32 μg/mL for intermediate, and ≥64 μg/mL for resistant [6,126]. An MIC > 64 μg/mL is generally associated with mutations in the 16S rRNA (*rrs*) gene [127]. Reported rates of amikacin resistance among MAC vary widely from 2% to >20% [99]. A 2025 retrospective review of 203 MAC respiratory isolates found that 1.5% (3/203) were phenotypically resistant to amikacin, with low prevalence of mutations on whole-genome sequencing [128]. A 2024 Spanish study found in vitro MAC amikacin resistance rates below 10% among 134 MAC isolates, with higher rates of aminoglycoside resistance observed among *M. avium* isolates than among *M. intracellulare* isolates [129]. In a 2023 Thai cohort, 36.8% of 38 MAC isolates were resistant to amikacin, with *rrs* mutations identified in resistant strains [130]. One study in China reported that 125/244 (51%) of *M. intracellulare* isolates were resistant in vitro to amikacin resistance [122].

Interestingly, not all MAC isolates with elevated MIC to amikacin may lead to worse clinical outcomes. A retrospective study in Ontario of 45 patients who had received IV amikacin found similar outcomes between 35 (77.8%) patients whose isolates had amikacin MIC < 64 mcg/mL, and 10 (22.2%) patients whose isolates had amikacin MIC ≥ 64 mcg/mL [131]. Sequencing of the *rrs* gene demonstrated that all study isolates had wild-type sequences [131]. 55% of MAC isolates in British Columbia tested between 2016 and 2020 were either intermediate or resistant to amikacin in vitro [121].

Rates of in vitro resistance to amikacin among rapidly growing NTM vary widely by species. One study generating an antibiogram for rapidly growing NTM pooled 3860 clinical isolates at a major reference laboratory in the United States [132]. Reported susceptibility to amikacin ranged from 82% in *M. abscessus* subspecies *massiliense* to 93% in *M. abscessus* subspecies *abscessus* and 100% in *M. fortuitum* [132]. A Chinese study found similarly high rates of amikacin susceptibility among MAB isolates—96.9% for *M. abscessus* subspecies *abscessus* and 100% for *M. abscessus* subspecies *massiliense* [124]. Generally, amikacin is the preferred aminoglyoside for treatment of rapidly growing mycobacterial infection. However, *M. chelonae* is more likely to be susceptible in vitro to tobramycin than to amikacin [68,88,133].

#### 5.2.3. Rifamycin Resistance

In vitro rifampin resistance has been observed in slow-growing mycobacteria, including MAC and *M. kansasii*. It is unclear what this means in vivo [134]. Unlike macrolides and amikacin, where in vitro susceptibility is correlated with better clinical outcomes, this is not the case for rifamycins in most slow-growing mycobacteria, including MAC. *M. kansasii* is a notable exception. While in vitro rifampin resistance remains relatively uncommon among *M. kansasii* isolates, when it occurs, it is generally due to *rpoB* gene mutations [134]. Rifampin resistance portends poor response of *M. kansasii* infection to rifampin-containing regimens, and an alternative regimen should be chosen in such cases [135,136,137]. In contrast, there is no correlation between in vitro rifampin susceptibility and treatment outcomes in MAC [138]. Acknowledging the uncertainty in clinical significance, in vitro resistance rates to rifampin among MAC can be as high as 60–70% [138]. Rifabutin, a rifamycin derivative, has been observed to have lower MICs than rifampin against slow-growing NTM species, suggesting it may potentially have more in vivo activity [139]. However, a meta-analysis comparing rifampin vs. rifabutin in MAC treatment found comparable treatment success rates between the two agents [140].

#### 5.2.4. Fluoroquinolone Resistance

Fluoroquinolones, particularly moxifloxacin, have a role in the treatment of some NTM species, particularly *M. xenopi* and *M. fortuitum* [6,68]. However, many NTM isolates are resistant in vitro to fluoroquinolones, which limits their utility in clinical practice. Although a number of mechanisms have been described, mutations conferring resistance to moxifloxacin are most commonly in the *gyrA* and *gyrB* genes which encode DNA gyrase [100,141]. Moxifloxacin resistance rates in a single-centre retrospective analysis of patients with macrolide-resistant MAC were 22% prior to moxifloxacin exposure and 67% after moxifloxacin exposure, although treatment success was not correlated with moxifloxacin susceptibility in this small study [142]. Another nationwide study in China found that while 0% of *M. kansasii* isolates were resistant to moxifloxacin, 47.22% of MAC isolates and 26% of other slowly growing mycobacteria were moxifloxacin resistant [143]. In this study, 90.63% of rapidly growing mycobacteria were resistant to moxifloxacin in vitro [143].

#### 5.2.5. Clofazimine Resistance

In vitro susceptibility breakpoints for clofazimine are not defined for NTM. However, clofazimine MIC can be determined for clinical isolates via the broth microdilution method and can be requested from the National Microbiology Laboratory in Canada. A meta-analysis of 20 studies reporting clofazimine MIC found pooled in vitro resistance rates of 9% in MAC and 16% in MAB [144]. In another study of 133 strains of MAC and 40 strains of MAB from 160 patients, clofazimine MICs for MAC ranged from 0.031 mg/L to 8 mg/L and for MAB from 0.031 mg/L to 16 mg/L [145]. 20 of these patients were treated with a clofazimine-containing regimen. Sputum conversion was seen in 8/20 patients, but sputum conversion was seen in all patients with clofazimine MIC ≤ 0.25 mg/L [145]. Clofazimine MIC ≤ 0.25 mg/L was significantly associated with culture conversion compared with patients with clofazimine MIC > 0.5 mg/L (OR, 39.3; *p* = 0.021) [145].

Overall, patterns of emerging resistance to these important antibiotics underscore the need for enhanced surveillance, improved diagnostic methods, and innovative treatment strategies. The integration of laboratory surveillance data with clinical insights is essential to address the complexities of NTM infections and their associated antimicrobial resistance effectively.

### 5.3. Challenges in Designing an Effective Regimen

#### 5.3.1. Species Diversity and Limited Evidence

With over 190 recognized NTM species and a lack of robust clinical evidence to guide therapy for less common organisms, clinicians may face uncertainties when designing treatment protocols. Outside of more well-studied organisms, regimen selection frequently relies on small case series or in vitro antimicrobial susceptibility testing, with uncertain interpretation due to the lack of clinically establishment susceptibility breakpoints [6,68].

#### 5.3.2. Empiric Versus Targeted Therapy

Unlike acute bacterial infections that require immediate empiric coverage, treatment of NTM disease can typically be delayed pending species identification and antimicrobial susceptibility testing results. Empiric treatment is rarely necessary [8]. In severe disseminated disease, however, initial empiric therapy may be justified, with close monitoring and prompt de-escalation once susceptibility results are available [6]. There can be barriers to accessing antimicrobial susceptibility testing. It is not immediate or routine and requires specific request and sample shipment to a specialized laboratory. Some NTM species may be challenging to culture in the laboratory making susceptibility testing inaccessible. Even when antimicrobial susceptibility testing is obtained, the lack of established clinical breakpoints for many of these drugs means MIC values do not always predict clinical success. To date, breakpoints are best defined for macrolides (and amikacin) in MAC and MAB, but efforts are ongoing to standardize criteria for newer agents, including bedaquiline against rapidly growing mycobacteria [146,147].

#### 5.3.3. Accessibility of Appropriate Antibiotics

While clinicians may be comfortable with medical management, many are less familiar with the complexities of acquiring special access drugs, navigating funding pathways, or coordinating with pharmacies for monitoring and documentation. For example, clofazimine and investigational agents such as bedaquiline require Health Canada Special Access Program approval, followed by coordination with manufacturers and community pharmacies—a process that can take 2–3 weeks. Amikacin liposome inhalation suspension (ALIS), despite its proven efficacy, is no longer available via Canada’s Compassionate Use Program. Now accessed through the Special Access Program on a chargeable basis (approximately $9600 US Dollars per 28-day kit), its cost and logistics make it inaccessible for most patients. As a result, many clinicians rely on nebulized preservative-free injectable amikacin, which in British Columbia requires special provincial authorization and is only covered for patients with Pharmacare coverage who have met their deductible (British Columbia formulary). The average 28-day cost of inhaled injectable amikacin is approximately $2200 CAD, excluding equipment. There are additional medications with utility in NTM therapy, including omadacycline and tedizolid, which are accessible in the United States of America but are generally not accessible in Canada.

In fact, in a 2018 survey of 36 healthcare providers from different health authorities in British Columbia, 61% cited medication access as the most common barrier to providing NTM care, followed by time constraints (58%) and lack of experience (39%) [148]. Limited familiarity with medication access processes can lead to delays, higher out-of-pocket patient costs, and decreased adherence, ultimately impacting treatment decisions and patient outcomes. A coordinated multidisciplinary approach is essential to navigate these barriers effectively.

#### 5.3.4. Therapeutic Drug Monitoring: Utility and Limitations in NTM Care

As NTM treatment regimens have evolved, the importance of individualized patient management has become increasingly evident. Recent studies have underscored the potential utility of therapeutic drug monitoring (TDM) and the role of tailored dosing strategies, especially with agents like macrolides and rifabutin, to optimize efficacy while minimizing adverse effects [90,149].

However, clinical application remains limited by interpretive challenges and extrapolated data [90,150]. The most common and evidence-based TDM practice in NTM care is amikacin trough monitoring, primarily used to assess nephrotoxicity, with a target trough <5 μg/mL, and preferably undetectable. Current American Thoracic Society/Infectious Diseases Society of America guidelines and expert opinion also suggest peak amikacin targets (e.g., 65–80 mg/L with thrice-weekly dosing), but these thresholds are derived from a single study that included mostly patients with tuberculosis and did not assess efficacy outcomes [151]. In fact, the original peak threshold was designed as a toxicity boundary, not an efficacy marker. Further complicating interpretation, local laboratory assays in BC cap detectable levels at 50 mg/L, reporting any higher value as a generic “critical” result, which limits clinical utility. For MAB, a Cmax/MIC ratio > 3 has been proposed, but this is only based on in vitro hollow fibre models [152]. More broadly, the absence of validated pharmacokinetic and pharmacodynamic targets and MIC breakpoints for many antibiotics in NTM, along with logistical barriers such as cost, assay availability, and the long half-lives of drugs like bedaquiline and clofazimine (which complicate dose adjustment), further restricts the routine implementation of TDM in NTM practice.

### 5.4. Treatment Completion Challenges

Even after choosing a patient-specific regimen, clinicians continue to face issues in implementing the regimen. Polypharmacy in typically older, frail patients leads to high rates of adverse events. Although detailed management strategies are outlined elsewhere [153], anecdotally, the Canadian guidelines suggest staggered drug initiation (adding one agent every 7–14 days), flexible dosing schedules (e.g., thrice-weekly versus daily) or changing within a class (clarithromycin to azithromycin or rifampin to rifabutin) to enhance tolerability and adherence without increasing risk of resistance [121]. Indeed, in non-cavitary MAC pulmonary disease, intermittent therapy with thrice weekly macrolide, rifampin and ethambutol was associated with better tolerance with no statistical difference in radiological, clinical or microbiological response [154].

Extended therapy brings cumulative toxicities—such as rifamycin-induced neutropenia and ethambutol-related optic neuropathy—as well as frequent monitoring burdens which may affect quality of life, and substantial cost [155,156,157,158]. Patient comorbidities, changing treatment preferences, limited options for resistant strains and incomplete treatment response rates may necessitate re-evaluation of therapeutic goals, prioritizing quality of life and symptom management over microbiological cure in some cases.

### 5.5. Infrastructure and Capacity for Multidisciplinary Care

Effective NTM-PD management relies on integrated multidisciplinary teams—including respirology and infectious disease specialists, clinical pharmacists, respiratory physiotherapists, microbiologists, nurses, and allied health professionals—working within a coordinated service framework. For extrapulmonary NTM, other specialties such as orthopedics or plastic surgery may also be required. Multidisciplinary clinics offer patients more holistic, coordinated, and long-term care, improving access to both diagnostic and therapeutic resources [151,159]. Across Canada, there remains a significant gap in the delivery of care for patients with NTM disease. While there are now a few multidisciplinary clinics available in select major centres, the infrastructure, resources, and expertise to support NTM disease care are still lacking in many regions.

## 6. Opportunities—Emerging Therapies and Precision Approaches in NTM-PD

Unlike TB, NTM treatment has historically lacked coordinated efforts and investment in drug development. However, the rising global burden of NTM disease and limitations of current regimens—such as high pill burden, prolonged duration, toxicity, and reliance on injectables—have renewed interest in novel therapies and repurposing existing therapies used for other mycobacteria such as MTBC. Bedaquiline is a diarylquinoline antibiotic that targets adenosine triphosphate synthase by binding to a subunit that is coded by the *atpE* gene. It was originally designed to treat multidrug-resistant TB but is now considered a promising agent in the treatment of NTM disease [147,160]. The evidence is stronger for bedaquiline in the treatment of extra-pulmonary NTM disease compared to pulmonary NTM disease [147]. However, bedaquiline utility in pulmonary disease due to MAC and MAB is being explored (Table 1).

Though the NTM pipeline remains limited, promising advances include new small molecules, re-engineered delivery systems, and non-traditional treatment approaches. In addition to novel systemic and inhalational therapeutic agents, there is growing interest in the role of biofilms in NTM persistence and progression and in the potential role of enzymatic biofilm disruption in enhancing the activity of antimicrobials with NTM activity [161,162,163,164,165].

Bacteriophage therapy is another emerging strategy for NTM treatment [166]. Methods of delivery include intravenous and inhalation, with some case reports of success and no reports of adverse reactions. Resistance of mycobacteria to bacteriophages has not been described [10]. However, host sensitization and the development of in vivo phage-neutralizing antibodies can occur. A larger issue with phage therapy is scalability. Currently, this approach is experimental, with attendant regulatory/import challenges. Additionally, therapy is individualized and requires substantial in vitro testing—the bacteriophage strain must show in vitro activity against the target NTM strain.

We highlight emerging NTM treatment strategies in three tables: Table 1 outlines novel approaches of previously approved therapies; Table 2 lists ongoing clinical trials; and Table 3 summarizes key preclinical agents and notable case reports. 

## 7. Conclusions

In conclusion, the four-decade evolution of NTM disease in Canada represents a complex interplay of improved recognition, changing epidemiology, therapeutic advances, and emerging challenges. While significant progress has been achieved in diagnosis and treatment, the rising prevalence of NTM disease and the emergence of difficult-to-treat species and antimicrobial resistance threaten these gains. The quiver of antibiotic treatments is limited, but new treatments are emerging. In part due to smaller markets, access to novel treatments in Canada is often not contemporaneous with their approval in other jurisdictions. Treatment is complex and prolonged, and clinical response rates are suboptimal. A multidisciplinary approach is critical. Because of limitations in treatment tolerability and efficacy and because of the crucial role of structural lung disease in NTM lung disease pathogenesis, antibiotics are just one approach to NTM disease treatment and should not be used in isolation or seen as a panacea. Airway clearance, nutritional optimization, immune restoration, and surgery, in some cases, are important pillars of NTM treatment and should be used in conjunction with antibiotics. Additionally, patients with NTM lung disease experience unacceptably high rates of relapse and reinfection and need ongoing surveillance and potentially multiple courses of therapy. The future of NTM disease management in Canada will depend on sustained investment in research, surveillance, and healthcare infrastructure. The development of novel therapeutic approaches, enhanced diagnostic capabilities, and improved understanding of disease pathogenesis will be essential for addressing emerging challenges. Most importantly, the complexity of NTM disease requires continued collaboration among clinicians, researchers, public health officials, and policymakers.

These parameters—from evolving NTM epidemiology and advancements in diagnostic tools, to the development of new treatment regimens and shifting antimicrobial resistance patterns—all point to significant dynamic changes in the Canadian NTM disease landscape. By exploring these trends, we can better understand the complexity of NTM disease management. There is a need for continued innovation and a multidisciplinary approach to NTM management.

## Figures and Tables

**Table 1:** Clinical trials for novel approaches to previously approved regimens.

Intervention	Comparator	Population	Trial Name	Status
Bedaquiline, clofazimine, linezolid + 2 or 3 drugs	N/A	Severe NTM-PD (defined based on CT) with macrolide-resistant or non-responsive MAC & MAB	NCT05494957 - Phase 4, Open label, Single group assignment	Not yet recruiting
2-drug regimen (macrolide + rifampin OR ethambutol)	3-drug regimen (macrolide + rifampin AND ethambutol)	Non-cavitary MAC-PD (*n* = 474)	NCT03672630 - Phase 2/3	In progress, estimated completion date October 2025
RHB-204 (fixed dose combination tablet: clarithromycin, rifabutin, and clofazimine	Placebo	Nodular-bronchiectatic MAC	NCT04616924 (CleaR-MAC), - Phase 3, randomized, double-blind, placebo-controlled	Terminated due to slow enrollment

Abbreviations: MAC—*Mycobacterium avium* complex; MAB—*Mycobacterium abscessus*; NTM-PD—Nontuberculous Mycobacterial Pulmonary Disease.

**Table 2:** Major Clinical Trials (ongoing or recently stopped).

Class	Intervention vs. Comparator	Proposed MOA	Population	Study ID/Phase	Core Findings
Aminobenzimidazole	SPR720 (fobrepodacin) vs. Placebo	Oral pro-drug → SPR719; GyrB ATPase inhibitor	MAC-PD, treatment naïve or off therapy at least 3 months (*n* = 25)	NCT04553406 - Phase 2 (suspended December 2024)	Interim analysis (*n* = 16) did not show sufficient separation from placebo. Safety concern with 3 cases of grade 3 hepatotoxicity
Aminomethylcycline (3rd generation Tetracycline)	Omadacycline vs. Placebo	Protein synthesis inhibition	Non-cavitary MAB-PD not on therapy (*n* = 66)	NCT04922554 - Phase 2, double-blind, randomized, parallel-group, placebo-controlled	34% of patients had improvement in at least half of their symptoms vs. 20% in placebo group (*p* value > 0.05). Omadacycline improved secondary efficacy outcomes (hypothesis generating) for symptom-based global impression endpoints [167].
Benzoxaborole	Epetraborole (EBO-301) + GBT vs. Placebo + GBT	Protein synthesis inhibition through inhibition of bacterial leucyl-tRNA synthetase	Refractory MAC-PD (*n* = 117)	NCT05327803 - Phase 2/3, double-blind, randomized, parallel-group, placebo-controlled (Terminated August 2024)	Truncated Phase 3 study (*n* = 97) misses primary endpoint; results unable to confirm clinical efficacy observed in Phase 2 study.
Biologics	Inhaled Molgramostim in addition to GBT or no treatment vs. (no comparator)	Unclear, likely stimulation of macrophage proliferation and function	Refractory NTM-PD (*n* = 32)	NCT03421743 (OPTIMA) Phase 2, prospective, open-label	8 patients (25%) achieved culture conversion which was sustained only in 4 of them. Main safety finding was eosinophilia [168].
Biologics	Recombinant Interleukin-7 (CYT107) vs. (no comparator)	Proposed to be immune activation	Refractory MAC-PD (*n* = 8)	NCT04154826 (IMPULSE-7) Phase 2, prospective, single-blinded	early termination due to futility. After treatment for 4 weeks, there was no culture conversion at 6 months and no change in symptoms [169].
Inhaled liposomal aminoglycoside	ALIS (liposomal amikacin) in addition to AZI + EMB for 6 months vs. Empty liposomal control in addition to AZI + EMB for 6 months	protein synthesis inhibition	New diagnosis non-cavitary MAC-PD or recurrent MAC-PD not receiving therapy	NCT04677543 (ARISE) - Phase 3, Randomized double blind, placebo-controlled	Addition of ALIS to standard therapy for 6 months led to higher culture conversion rates by Month 6 (80.6% vs. 63.9%) and Month 7 (78.8% vs. 47.1%) vs. comparator. (*p* = 0.001). No resistance to amikacin or macrolide developed; recurrence was significantly lower with ALIS (12.8% vs. 50%) and occurred in the absence of resistant emergence [170].
Inhaled liposomal aminoglycoside	ALIS (liposomal amikacin) in addition to AZI + EMB for 12 months vs. Empty liposomal control in addition to AZI + EMB for 12 months	protein synthesis inhibition	New diagnosis non-cavitary MAC-PD or recurrent MAC-PD not receiving therapy	NCT04677569 (ENCORE) - Phase 3, Randomized double blind, placebo-controlled	Study in progress. This is the confirmatory study for ARISE to evaluate safety/efficacy at month 13 [1 month off treatment] as well as % with durable culture conversion at month 15 [3 months off treatment].
Metal-based compounds	Intravenous Gallium nitrate in addition to GBT or no treatment vs. (no comparator)	Iron substitution and interruption of bacterial metabolism	Refractory NTM-PD in CF patients (*n* = 40)	NCT04294043 (ABATE), - Phase 1, safety study, prospective	Study in progress
Nitric oxide	Intermittent inhaled nitric oxide vs. (no comparator)	Oxidative and nitrosative stress induction	NTM-PD in CF and non-CF (*n* = 10)	NCT03748992 - Phase 2, single group assignment,	4 out of 10 patients achieved negative sputum culture within 3 weeks but were positive again within 3 months post therapy) [171].
Oxazolidinone	Delpazolid in addition to GBT vs. (no comparator)	Protein synthesis inhibition	Refractory MAB-PD (*n* = 20)	NCT06004037 - Phase 2, prospective	Study in progress
Personalized bacteriophages	Phage cocktails in addition to GBT vs. (no comparator)	Lytic phages lyse Mab cell wall	Refractory MAB in CF patients (*n* = 10)	NCT06262282 (POSTSTAMP) Prospective, single group assignment	Enrolling phase
Riminophenazine	Clofazimine loading dose in addition to GBT vs. (no comparator)	Cell replication disruption and ROS generation through binding Mycobacterial DNA	Non-CF extrapulmonary or pulmonary NTM disease and weight > 45 kg (*n* = 12)	NCT05294146 (C-LOAD) - Phase 2, PK study, open label, single group assignment	4-week loading dose regimen of 300 mg once daily reduced the time to target clofazimine concentrations by ~1.5 months and was well tolerated with GI symptoms as main side effect [172].
Riminophenazine	Inhaled clofazimine in addition to GBT vs. Placebo in addition to GBT	Cell replication disruption and ROS generation through binding Mycobacterial DNA	MAC-PD currently on GBT	NCT06418711 (ICoN-1) - Phase 3, randomized, double-blind, placebo-controlled	Study in progress
Tetracycline	Minocycline in addition to rifampin and other GBT vs. (no comparator)	Protein synthesis inhibition through 30S ribosomal subunit	Non-CF MAC-PD (planned *n* = 15)	NCT05861258 (Mino-PK) - Phase 2, PK study, single group assignment	Recruiting

Abbreviations: ALIS—Amikacin Liposome Inhalation Suspension; AZI—Azithromycin; CF—Cystic Fibrosis; EMB—Ethambutol; GBT—Guideline-Based Therapy; MAC-PD—*Mycobacterium avium* complex pulmonary disease; MAB-PD—*Mycobacterium abscessus* pulmonary disease; MOA—Mechanism of Action; NTM-PD—Nontuberculous mycobacterial pulmonary disease; PK—Pharmacokinetics; ROS—Reactive Oxygen Species.

**Table 3:** Major pre-clinical, case studies, or population-based cohort.

Class	Compound Name	Proposed MOA	Notable Studies	Notable Findings
Benzothiazinone	Macozinone (PBTZ169, MCZ)	Cell wall synthesis inhibition	*M. avium*, MAB, and *M. fortuitum* clinical strains|MAB, *M. chelonae*, and *M. fortuitum* mouse models	Despite high MICs in vitro, it was bactericidal against *M. abscessus & M. chelonae* and bacteriostatic against *M. avium & M. fortuitum* in the lung and spleen of murine model Also, under development for TB, completed phase 1 safety trials [173,174].
Benzoxaborole	MRX-5 (oral prodrug for MRX-6038)	Protein synthesis inhibition through inhibition of bacterial leucyl-tRNA synthetase	MAB in mouse model|Healthy human subjects for PK study	Phase 1 clinical trial in Australia showed no SEs (unpublished data). Undergoing phase 2 in China; FDA Orphan Drug Designation granted in December 2024 [175,176].
Biguanide (host-directed)	Metformin	AMPK activation → autophagy & ROS ↑ Enhance macrophage activity	*M. avium* mouse model Cohort of patients in a large population-based study	Mouse lung bacterial load ↓; macrophage killing ↑ ↓ hazard of incident NTM infection by 62.4% in a cohort of pt with MAC disease also on metformin [177,178].
Diarylquinoline	Sudapyridine (WX-081)	BDQ analogue (ATP-synthase blockade), ↓ QTc prolonging based on studies in tuberculosis [179].	Clinical isolate *M. avium* and MAB|Intracellular MAB in macrophages|Immunocompromised mouse model infected with *M. avium*, MAB or *M. chelonae* [undergoing phase 2 for MDR-TB]	Bactericidal in vitro in all three strains Intracellular antimicrobial activity comparable to BDQ [180].
Diarylquinoline	TBAJ-876 (3,5-dialkoxypyridine analogue of BDQ)	BDQ analogue, ↓ lipophilicity	MAB reference strains|MAB clinical isolates|Intracellular MAB in macrophages|immunocompromised mouse model	Bactericidal activity in vitro Intracellular antimicrobial activity comparable to BDQ Clinical efficacy in mouse model comparable to BDQ Compatible with other NTM meds [181].
Dual beta-lactam +/− beta-lactamase inhibitor	Different combinations based on in vitro synergy testing mostly carbapenems + cephalosporins (e.g., ceftaroline + imipenem or ceftazidime + imipenem) +/− relebactam or avibactam (e.g., Imipenem/cilastatin + relebactam + amoxicillin)	Disruption of cell envelope through inhibition of both D, D-transpeptidases and L, D-transpeptidases Beta-lactamase inhibitors increase activity of beta-lactam	MAB complex In vitro|mouse models|case studies in treatment refractory MAB-PD	Synergistic effect against MAB in vitro and in vivo (although very limited data). Patient cases suggest a higher risk of elevated liver enzymes. No current clinical trial [182,183].
Oxazolidinone	Contezolid (MRX-I)	inhibits bacterial protein synthesis, ↓ hematologic and neurologic toxicity compared to linezolid	MAB complex In vitro strains|zebrafish Compassionate use in MAB SSTI unable to tolerate linezolid	Inhibited MAB growth and prolonged zebrafish survival. Successful treatment of MAB SSTI with no adverse events reported after 6 months [184,185].
Oxazolidinone	tedizolid	inhibits bacterial protein synthesis	Invitro laboratory and clinical isolates|single-centre retrospective cohort in solid organ transplant recipients (*n* = 15 tedizolid, 9 linezolid)	Efficacy reported against MAC with a lower MIC compared to linezolid in in vitro research. Synergy with ethambutol reported [186,187]. Retrospective data in solid organ transplant recipients showed similar efficacy and safety compared to linezolid over 7 weeks of therapy [188].
Tetracycline	Eravacycline (intravenous)	Protein synthesis inhibition	retrospective, single-centre cohort of 16 patients	Despite claims of clinical improvement, nearly half discontinued ERV due to adverse events and only 50% completed OPAT. Results are limited by short treatment duration and unclear what clinical improvement entailed as primary efficacy outcome [189].

Abbreviations: AMPK—AMP-activated protein kinase; ATP—Adenosine Triphosphate; BDQ—Bedaquiline; ERV—Eravacycline; In vitro—Outside a living organism (e.g., lab culture); In vivo—Within a living organism (e.g., animal models); MAC—*Mycobacterium avium* complex; MAB—*Mycobacterium abscessus*; MAB-PD—*Mycobacterium abscessus* pulmonary disease; MDR-TB—Multidrug-resistant tuberculosis; MIC—Minimum Inhibitory Concentration; MOA—Mechanism of Action; OPAT—Outpatient Parenteral Antimicrobial Therapy; PK—Pharmacokinetics; ROS—Reactive Oxygen Species; SSTI—Skin and Soft Tissue Infection.

## Data Availability

No new data were created or analyzed in this study. Data sharing is not applicable to this article.
